# Factors influencing participation in a vascular disease prevention lifestyle program among participants in a cluster randomized trial

**DOI:** 10.1186/1472-6963-13-201

**Published:** 2013-05-31

**Authors:** Rachel A Laws, Mahnaz Fanaian, Upali W Jayasinghe, Suzanne McKenzie, Megan Passey, Gawaine Powell Davies, David Lyle, Mark F Harris

**Affiliations:** 1 Prevention Research Collaboration, School of Public Health, University of Sydney, Sydney, NSW 2006, Australia; 2 Centre for Primary Health Care and Equity, School of Public Health and Community Medicine, University of New South Wales, Sydney, NSW 2060, Australia; 3 School of Medicine and Dentistry, James Cook University, Townsville, QLD 4811, Australia; 4 University Centre for Rural Health- North Coast, School of Public Health, University of Sydney, Sydney, NSW 2006, Australia; 5 Broken Hill University Department of Rural Health, School of Public Health, University of Sydney, Sydney, NSW 2006, Australia

**Keywords:** Preventive health care, Lifestyle modification, Attendance rates, Reach, Primary care, Family practice, Chronic disease prevention

## Abstract

**Background:**

Previous research suggests that lifestyle intervention for the prevention of diabetes and cardiovascular disease (CVD) are effective, however little is known about factors affecting participation in such programs. This study aims to explore factors influencing levels of participation in a lifestyle modification program conducted as part of a cluster randomized controlled trial of CVD prevention in primary care.

**Methods:**

This concurrent mixed methods study used data from the intervention arm of a cluster RCT which recruited 30 practices through two rural and three urban primary care organizations. Practices were randomly allocated to intervention (n = 16) and control (n = 14) groups. In each practice up to 160 eligible patients aged between 40 and 64 years old, were invited to participate. Intervention practice staff were trained in lifestyle assessment and counseling and referred high risk patients to a lifestyle modification program (LMP) consisting of two individual and six group sessions over a nine month period. Data included a patient survey, clinical audit, practice survey on capacity for preventive care, referral and attendance records at the LMP and qualitative interviews with Intervention Officers facilitating the LMP. Multi-level logistic regression modelling was used to examine independent predictors of attendance at the LMP, supplemented with qualitative data from interviews with Intervention Officers facilitating the program.

**Results:**

A total of 197 individuals were referred to the LMP (63% of those eligible). Over a third of patients (36.5%) referred to the LMP did not attend any sessions, with 59.4% attending at least half of the planned sessions. The only independent predictors of attendance at the program were employment status - not working (OR: 2.39 95% CI 1.15-4.94) and having high psychological distress (OR: 2.17 95% CI: 1.10-4.30). Qualitative data revealed that physical access to the program was a barrier, while GP/practice endorsement of the program and flexibility in program delivery facilitated attendance.

**Conclusion:**

Barriers to attendance at a LMP for CVD prevention related mainly to external factors including work commitments and poor physical access to the programs rather than an individuals’ health risk profile or readiness to change. Improving physical access and offering flexibility in program delivery may enhance future attendance. Finally, associations between psychological distress and attendance rates warrant further investigation.

**Trial registration:**

ACTRN12607000423415

## Background

Cardiovascular disease (CVD) and diabetes contribute significantly to global disease burden. In Australia, CVD and diabetes are the leading causes of health loss (disability adjusted life years) [1]. Risk factors for CVD such as obesity, diabetes, hyperlipidaemia and hypertension often cluster together and have a significant negative impact on quality of life [2].

The efficacy of intensive lifestyle interventions in preventing diabetes and CVD amongst high risk individuals has been well established in a number of large randomised controlled trials [3-9]. Meta-analyses of randomized controlled trials have shown that lifestyle intervention can reduce the incidence of diabetes by around 50% [10] and is at least as effective as drug treatment [11]. Key features of these successful interventions include individual or group counseling sessions to improve diet and physical activity, multiple contacts with participants over an extended period of time (at least 6 months) and supervised exercise sessions [6,8,12].

Translating the findings from successful randomized controlled trials to achieving health benefits at the population level depends upon the reach and efficacy of lifestyle interventions when delivered as part of routine service provision. Research published to date has focused largely on the efficacy of lifestyle interventions in the prevention of diabetes and CVD when delivered in highly controlled clinical trials. In these trials, recruitment rates are typically low, hence those recruited tend to be a highly selective group of motivated individuals resulting in high levels of participation in intervention programs. For example, in the US Diabetes Prevention Program, only 2.4% of those initially screened were randomized to participate in the trial [13], however intervention participation amongst those randomized was high [8].

Recent implementation trials examining the feasibility of delivering lifestyle intervention programs in primary health care and community settings have demonstrated that it is possible to implement lifestyle intervention programs in real world settings and results are promising [14-20]. However rates of attendance and completion of the lifestyle programs in these studies varied greatly, ranging from 50 to 80% with little information provided on the factors affecting attendance and completion [14,18,19,21]. Some studies did report that completers were more likely to be older [14], have higher levels of education [18], lower levels of psychological distress [18], and higher perceived disease risk factor compared to non completers [22]. While the definition of completion varied across studies, a dose response was found between attendance rates and changes in lifestyle and physiological risk factors [18,19]. This underscores the importance of understanding the factors which affect individual engagement in such programs through quantitative and qualitative analysis.

The Health Improvement and Prevention Study (HIPS) [23] was an Australian implementation trial that aimed to evaluate the impact of a general practice based intervention for individuals at risk of vascular disease on changes in behavioural and physiological risk factors. In this study we found that individuals who attended their GP for a health check were able to achieve changes in their self-reported physical activity behaviours, however, only those who were referred to and attended a lifestyle modification program (LMP) achieved a significant improvement in diet or weight [24].

This paper reports a secondary analysis of data from HIPS to explore factors influencing the level of participation in the LMP delivered as part of this randomized controlled trial of CVD prevention in general practice. This will help inform how better to engage high risk individuals in lifestyle modification programs, particularly in the primary health care setting.

## Methods

This paper analyses data collected as part of a larger trial, the Health Improvement and Prevention Study (HIPS), the details of which have been published elsewhere [23].

### Recruitment

The study was conducted in two rural and three urban Divisions of General Practice (Primary Care Organizations) in New South Wales (NSW) Australia. A total of 36 practices were invited to participate in the trial and 30 agreed to take part. Practices were randomly allocated to an ‘intervention’ (n = 16) or ‘control’ group (n = 14). Eligibility included having attended the practice in the previous 12 months, being aged 40–55 with hypertension and / or hyperlipedaemia or aged 56–64, and not being involved in other research. In each practice up to 160 eligible individuals were invited to participate by mail. Individuals were excluded if they had diabetes, cardiovascular disease, current severe illness, or were unable to speak adequate English or understand the consent form.

### Intervention

General practitioners (GPs) and practice nurses from intervention practices were offered a three hour training session on lifestyle assessment and counselling including brief motivational interviewing supplemented with practice visits and educational resources. Participating individuals were invited to attend for a health check during which the GP and practice nurse provided brief lifestyle counselling based on the 5As model (ask, assess, advise, assist, and arrange) [25]. In intervention practices, providers were encouraged to refer high risk individuals (defined as one or more of the following: history of gestational diabetes, impaired glucose tolerance or impaired fasting glycaemia, hypertension, hyperlipidaemia, body mass index >28 or waist circumference >102 cm in males or 88 cm in females, current smoker) to a LMP. The LMP was coordinated by trained Intervention Officers at local Primary Care Organizations. The program consisted of an initial visit with a dietitian or exercise physiologist, who conducted an assessment and negotiated individual dietary and physical activity goals with the participant and an individual review session with the same allied health professional following the group program. The group program, which was adapted from the group component of the “*Counterweight Program – CHANGE*” [26], consisted of four group sessions (1.5 hours each) over the first three months, and a further two follow up sessions at six and nine months. The group sessions included an educational and physical activity component (20–30 minutes of walking or resistance exercise) and were based on the use of self-management strategies (goal setting, self monitoring, developing practical skills and problem solving) to promote positive dietary and physical activity changes and weight loss. Between sessions participants were encouraged to keep a food and physical activity diary, use a pedometer and carry out home based physical activity. The program was offered out of business hours in some Divisions. Some urban Divisions also gave taxi vouchers to participants to provide transport to and from the sessions.

### Data collection

This paper used a concurrent mixed methods approach [27], drawing on both quantitative and qualitative data to explore factors influencing attendance rates at the LMP. Data sources [23] included:

1) Participant survey data

2) Participant clinical audit data

3) Practice questionnaire on capacity for preventive care

4) Semi-structured interviews and journal notes from Intervention Officers

5) Lifestyle modification program referral and attendance records

#### Participant survey data

Participating individuals completed a mailed survey at baseline, six and 12 months. The survey was based on the NSW Health Survey [28] and previous research [29,30]. It included questions about: (1) practice attendance; (2) reported assessment and management of lifestyle risk factors (smoking, nutrition, alcohol, physical activity and weight) and satisfaction with intervention received; (3) attendances at other services as a result of referral from the practice or self-referral; (4) self-reported fruit and vegetable intake, smoking, physical activity and alcohol intake, and attempts to change these; (5) readiness for behaviour change (stage of change) for each lifestyle risk factor [31]; (6) The Kessler Psychological Distress Scale (K-10), a ten item questionnaire measuring negative emotional states in the preceding four weeks [32], and demographic variables (age, gender, postcode of residence, education level, employment status, language spoken at home and country of birth).

The 12 month survey also included the Porter Novelli’s 10-item scale [33] which categorizes individuals into four distinct groups based on differences in degree of engagement in health enhancement (active versus passive) and degree of independence in health decision making (independent versus doctor dependent). Self-reported LMP attendance rates and reasons for non attendance were collected in the 12 month survey.

The postcode of residence for each participant was linked to the 2006 index of relative socio-economic advantage/disadvantage [34] for the area in which the participant lived. The index ranks geographical areas where a high proportion of people are relatively more, or less, disadvantaged taking into account income, education, occupation, wealth and living conditions. The index was linked to the participants’ postcode of residence using quintiles. A quintile number of one represented the lowest 20% of areas, up to the highest 20% of areas which were given a quintile number of five. For the purposes of analysis three categories were created: 1) most disadvantaged participants (quintiles one and two), 2) intermediate disadvantaged participants (quintile three) and 3) least disadvantaged participants (quintiles four and five).

#### Participant clinical audit data

GPs and practice nurses were requested to record participant weight, waist circumference and blood pressure at baseline and 12 months. These data were then extracted from patient records by trained data collectors. Participants were asked to have a fasting blood test to assess their serum lipids (total cholesterol, HDL, LDL, triglycerides) and glucose at baseline and 12 months and a copy of these results was sent directly to the study centre by the pathology company.

#### Practice questionnaire on capacity for preventive care

The practice manager or principal GP was asked to complete a questionnaire on practice capacity for preventive care. The questionnaire included questions on practice characteristics (location, size, employment of practice nurses), the use of education materials, staff roles and teamwork in preventive care as well as the use of written preventive care protocols and linkages between the practice and support services [35].

#### Semi-structured interviews and journal notes from Intervention Officers

All Intervention Officers (n = 5, two from rural and three from urban areas) were invited to participate in a semi-structured interview at the completion of the intervention. The interviews explored their experience of implementing the LMP, including the referral process, participant engagement and attendance, program content and process of delivery, participant outcomes and program sustainability. Interviews were carried out by a researcher not involved in the study. Interviews were conducted over the phone and recorded with participants’ permission and generally lasted between 20 and 45 minutes. Intervention Officers were also asked to keep a journal throughout the intervention period recording their reflections about the program.

#### Lifestyle modification program referral and attendance records

The Intervention Officers monitored and kept the records of GP referrals and attendance at the LMP. They were responsible for making initial contact with participants, scheduling the group program and individual allied health visits and monitoring participant attendance.

### Quantitative data analysis

#### Referral and attendance data

Data were initially subject to preliminary descriptive analysis using SPSS statistical software (version 20; SPSS, Chicago, IL, USA) to examine the frequency of attendance at the program. Individuals were then categorized according to their participation: as 1) non attender (did not attend any individual or group sessions), 2) low attender (attended less than half of all group/individual sessions) or 3) high attender (attended at least half of all individual/group sessions).

#### Characteristics of high versus low or non attenders

Univariate analysis was initially used to compare high attenders versus low/non attenders in terms of demographic characteristics, individuals health risk profile, health seeking behavior and readiness to change, degree of psychological distress, as well as previous GP intervention or referral for diet and physical activity. The characteristics of practices from which high and low/non attenders were referred were also compared. Significant differences in categorical variables for high and low/non attenders were examined using chi square statistic and independent samples *t*-test for normally distributed variables.

#### Factors influencing attendance

Variables found to be significant (p < 0.05) in the univariate analysis were entered into a multivariate logistic regression model to examine the predictors of high attendance at the LMP. The data were then subject to multi-level logistic regression analysis to examine participant and practice factors associated with attendance levels. The multi-level analysis was considered appropriate as participant data for attendance was clustered by practice (ICC = 0.111). The intra class correlation (ICC) represents the degree to which participant data from the same practice are similar to one another compared with those of individuals from different practices. The high ICC values indicate that the analysis must account for the variance between practices, supporting the choice of multi-level analysis [36]. Multilevel logistic regression models were used with a dichotomous dependent variable (0 = non/low attendance, 1 = high attendance) adjusted for clustering of individuals (level 1) and within practices (level 2) [37]. Initially, we fitted a baseline variance component or empty model (no independent variables) followed by the model with individual and practice variables (Model 1). ICC was calculated using the latent variable method. The (standard) logistic distribution has variance π^2^/3 = 3.29 and hence this can be taken as the level 1 variance [34]. As both the level 1 and 2 variances are on the same scale, the following formula was used: ICC = (level 2 variance)/(level 2 variance + 3.29). All multi-level models were performed with MLwiN version 2.0. [37].

### Qualitative data analysis

All interview data were transcribed verbatim and journal notes typed in a Word document. Factors influencing participant engagement and attendance at the LMP were then coded using NVivo 7.0 software [QSR [38] and key themes identified along with supportive quotes. All analysis was undertaken by one researcher (RL) with codes and themes discussed with the broader research team.

## Ethics

The study was approved by the UNSW Human Research Ethics Committee. All participants gave their informed consent to participate in the study.

## Results

### Attendance rates

**Figure 1:**
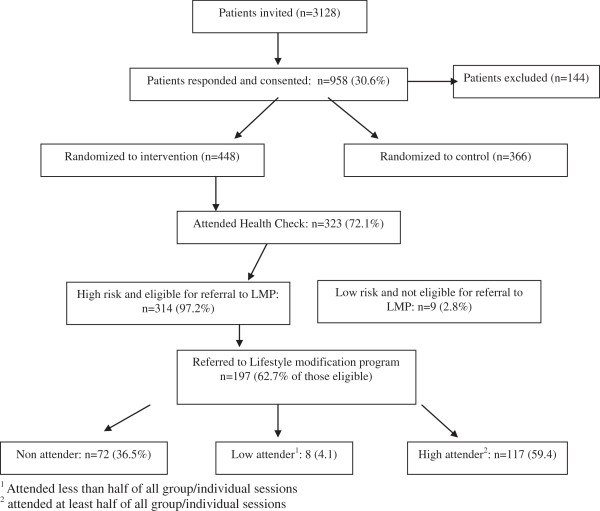
Patient recruitment and attendance at Lifestyle Modification Program (LMP).

A total of 197 individuals (63% of those eligible after attending the baseline health check) were referred to the LMP. Of these individuals, over one third (36.5%, n = 72) did not attend any of the individual or group sessions, eight attended less than half of the sessions (low attenders) and the remainder (n = 117, 59.4%) were high attenders, participating in at least half of all the group/individual sessions (Figure 1). For the purpose of further analysis low and non attender groups were combined (n = 80, 40.6%) and compared to the high attender group (n = 117, 59.4%).

### Characteristics of high versus low/non attenders

High attenders were significantly more likely to be not working (retired or not working due to other reasons), living in areas of moderate or high deprivation and to have higher levels of psychological distress compared to low/non attenders (Table 1). High attenders were also more likely to be from smaller and medium sized practices, practices that employed or rented rooms to allied health professionals and those with written preventive care protocols. There were no differences between high and low/non attenders in terms of other demographic and practice characteristics, health risk profile, health seeking behavior, readiness to change (Table 2) or previous GP intervention or referral for diet and physical inactivity (Table 3).

**Table 1:** Characteristics of high attenders compared to low/non attenders of the lifestyle modification program

	**High attenders**	**Low/non attenders**	**Significance**
	**n = 117**	**n = 80**	
**Demographic Characteristics**			
Female	78 (66.7%)	46 (57.5%)	P = 0.191
Mean age (St Dev)	58.2 (5.7)	57.3 (5.6)	P = 0.778
Age 40-54	25 (21.4%)	18 (22.5)	P = 0.850
Age 55-64	92 (78.6)	62 (77.5)	
Low deprivation area	25 (21.4%)	34 (42.5%)	P = 0.001*
Moderate deprivation area	70 (59.8)	42 (52.5)	
High deprivation area	22 (18.8)	4 (5.0)	
Education: TAFE/University/other qualification	64 (56.6%)	50 (62.5%)	P = 0.414
Employed	67 (58.8%)	62 (77.5%)	P = 0.007*
Retired	23 (20.2%)	7 (8.8%)	
Not working (other reasons^1^)	24 (21.1%)	11 (13.8%)	
Primarily speak English at home	99 (85.3%)	68 (85.0%)	P = 0.947
Australian Born	87 (75.0%)	58 (72.5%)	P = 0.743
**Patient Health Risk Profile**			
Blood pressure (>135/85)	14 (14.4%)	4 (7.1%)	P = 0.178
Lipids (LDL > 2, HDL < 1, TG > 1.5 or TC >4)	114 (97.4%)	74 (97.4%)	P = 0.977
BMI (≥25)	85 (73.3%)	61 (76.3%)	P = 0.639
Diet risk	43 (38.4%)	33 (45.8%)	P = 0.317
Physical inactivity	75 (64.7)	49 (62.0)	P = 0.708
Alcohol risk	31 (33.3%)	28 (44.4%)	P = 0.160
Tobacco	10 (8.8%)	9 (11.4%)	P = 0.561
Health status excellent/very good/good	99 (85.3)	65 (81.3)	P = 0.446
Psychological distress (K10score 16+)	49 (45.4%)	21 (27.3%)	**P = 0.012**
**Practice Characteristics / Factors**			
Urban practice	23 (19.7%)	22 (27.5%)	P = 0.198
Rural practice	94 (80.3%)	58 (72.5)	
Patient of practice for > 6 years	66 (61.7%)	36 (55.4%)	P = 0.577
Small practice (1–2 GPs)	50 (42.7%)	26 (32.5%)	P = 0.004*
Medium practice (3–4 GPs)	50 (42.7%)	26 (32.5%)	
Large practice (5+ GPs)	17 (14.5%)	28 (35.0%)	
Practice nurse(s) work at the practice	77 (65.8%)	60 (75.0%)	P = 0.169
Practice nurse(s) has a role in preventive care	60 (52.6%)	51 (65.4%)	P = 0.242
Administrative staff have a role in preventive care	104 (88.9%)	71 (88.8%)	P = 0.976
Practice employs or rents rooms to allied health professionals	88 (75.2%)	48 (60%)	P = 0.023*
Practice has written preventive care protocols	85 (72.6%)	52 (65.0%)	P = 0.002*
Provision of PA or diet advice/referral for those at risk (quality of care indicator)	49 (45.4)	27(35.5)	P = 0.182
Satisfaction with GP provision of diet and PA advice/referral for those at risk	60 (55.6)	34 (44.7)	P = 0.148

*P < 0.05,^**1**^ unemployed, in full time education, unable to work due to long-term sickness/disability, home duties, other.

**Table 2:** Health information seeking behavior and readiness to change for high versus low/non attenders (at Baseline)

	**High attenders**	**Low/non attenders**	**Significance**
**Categories:**			
Doctor-Dependent Active	0 (0%)	0 (0%)	P = 0.598
Doctor- Dependent Passive	40 (38.5%)	26 (42.6%)	
Independent Active	0 (0%)	0 (0%)	
Independent Passive	65 (61.5%)	35 (57.4%)	
Contemplation/preparation/action stage of change for increasing fruit and vege	52 (47.7%)	41 (51.3%)	P = 0.268
Contemplation/preparation/action stage of change for decreasing dietary fat intake	63 (57.8%)	46 (58.2%)	P = 0.823
Contemplation/preparation/action stage of change for doing more physical activity	83 (72.8%)	51 (64.6%)	P = 0.401
Contemplation/preparation/action stage of change for drinking less alcohol	33 (43.4%)	25 (44.6%)	P = 0.346
Contemplation/preparation/action stage of change for quitting smoking	12 (44.4)	7 (33.3)	P = 0.148
Contemplation/preparation/action stage of change for losing weight	79 (71.2)	54 (70.1)	P = 0.857

**Table 3:** Previous GP Intervention or referral for diet and or physical activity intervention and levels of satisfaction

	**High attenders**	**Low/non attenders**	**Significance**
GP nutrition intervention in previous 3 months	41 (42.3)	25 (41.7)	P = 0.941
GP physical activity intervention in previous 3 months	44 (45.8)	26 (42.6)	P = 0.693
Very satisfied with GP support for lifestyle change	36 (37.5)	25 (44.6)	P = 0.703
Very satisfied with support from ‘other services’ for lifestyle change	9 (15)	7 (20.6%)	P = 0.703

### Independent predictors of attendance rates

In multilevel multivariate analysis, controlling for clustering by practice and confounding co-variates, factors independently associated with high attendance were not being in employment (OR = 2.39, CI = 1.15-4.94) and high levels of psychological distress (OR = 2.17, CI = 1.10-4.30). Factors included in the model explained 94.4% of the variance between practices (Table 4).

**Table 4:** Multi-level logistic regression models for high attendance at lifestyle modification program

**Explanatory variables**		**Empty model**	**Model 1**^**1**^
**Participant Characteristics**		OR (95% CI)	OR (95% CI)
SEIFA index^2^	Least disadvantaged		1.00 (reference)
	Intermediate disadvantage		1.24 (0.35-4.32)
	Most disadvantaged		2.17 (0.21-22.15)
Employment status	Working		1.0 (reference)
	Not working^3^		2.39 (1.15-4.94)*
Psychological distress	Low/moderate distress		1.00 (reference)
(K10 score: 16+)	High distress		2.17 (1.10-4.30)*
**Practice Characteristics**			
Practice size	Large practice (5+ GPs)		1.00 (reference)
	Medium practice (3–4 GPs)		1.73 (0.60-5.05)
	Small practice (1–2 GPs)		3.46 (0.79-15.03)
Practice employs or rents rooms to allied health professional	No		1.00 (reference)
	Yes		2.24 (0.34-14.67)
Practice has written protocol for preventative care	No		1.00 (reference)
	Yes		1.60 (0.33-7.83)
Between practice variance (SE^3^)		0.410 (0.277)	0.023 (0.132)
Intra class correlation		0.111	0.007
Explained variance ^4^ (%)		-	94.4%

*P < 0.05.

Multilevel logistic regression^1^ Model 1: includes all variables found to be significant in univariate analysis. ^2^ 2006 index of relative socio-economic advantage/disadvantage, ^3^ Standard error, ^4^ Explained ‘between practice variance using the variance in the empty model as reference.

### Reasons provided by individuals for not attending the lifestyle modification program

Most of the non attenders (55.6%) did not provide any reason in the 12 month survey for not attending the program. Where reasons were given, work commitments was the most commonly cited (12.5%) followed by unsuitable date or time of the program or other commitments (Table 5).

**Table 5:** Patient reasons for not attending the lifestyle modification program

**Reason for not attending**	**Non attenders (n = 72) ** **No (%)**
Work commitments	9 (12.5)
Session date/time not suitable	5 (6.9)
Other commitments	5 (6.9)
Health issues	4 (5.6)
Lack of perceived need	2 (2.8)
Not enough notice	2 (2.8)
Program type	1 (1.4)
Other	4 (4.6)
No reason provided	40 (55.6)

### Factors influencing attendance – Intervention Officer perspective

Analysis of Intervention Officer interviews and journal notes highlighted a number of factors influencing participant attendance and engagement with the program (Table 6). For encouraging attendance, GP and practice staff endorsement of the program was identified as important, as was encouragement from the Intervention Officer when contacting participants to enroll them in the program.

**Table 6:** Factors influencing attendance – Themes identified from intervention officer interviews/journal notes

**Theme / Description**	**Illustrative quotes**
**Program Endorsement: ** Endorsement and encouragement to attend by GPs, PNs and Intervention Officers	*“Basically their GP had said to them “we’re, I really think that you should partake in this program” and it was just a, a process of actually…once I got them on the phone basically pretty much talking them into it” (IO5- metro site)*
**Access: ** including physical access (transport/parking), session times and flexibility in program delivery	*“Being an urban area, many people rely on alternative transport such as public transport. This barrier was overcome by offering people who did not drive (or who could not drive at night) cab-charges to the sessions” (IO5- metro site).*
	*“Geographical isolation was a huge issue for patients especially those on properties out of the area” (IO1 – rural site).*
	*“people aged 40 to 59 they’re generally working… There’s not many who aren’t…so you’re trying to get them to come to these sessions at night…And they’ve been at work all day…”(IO5- metro site).*
	*“I did after hours sessions for people who worked. I did different days of the week to suit all different patients…*
	*I’d try and catch up, do one on ones or if I couldn’t do that I’d chat with them over the phone, send them information and follow up” (IO1- rural site).*
**Patient factors:** gender, age and working status, patient risk perception and motivation and weight status	*“I would say the female participants seemed to work better....Take it on board …I couldn’t really compare it because …I didn’t have enough males participating” (IO3 – metro site).*
	*“you have a lot more luck talking to people who were …retired or semi retired” (IO5- metro site).*
	*“[attendance was better amongst ] the older participants....I guess it, it was more apparent that they needed to make changes otherwise they were going to shorten – their life span…because …they’re overweight or sedate” (IO3- metro site).*
	*“Patients with large BMIs (greater then 35) tended to not participate well in the walking or exercise section of CHANGE sessions” (Journal notes- IO2 rural site).*
**Social factors:** partner attendance, social interaction and group member familiarity and group leader facilitation skills	*“Look a lot of people wouldn’t have come unless they could bring their partner along” (IO5- metro site).*
	*‘Cause I presented it as if I was taking part in the program as well…– I had my own pedometer… did the walks myself and modified my diet as well....we had a very good rapport amongst ourselves....I think taking the time to develop that was an essential part of a good outcome rather than being sort of …there’s the, the teacher and the students (IO3 – metro site).*

A number of issues were identified in relation to participant access to the program. In urban areas, many people relied on public transport to access the group. People were reluctant to use public transport for sessions run in the evening for safety reasons. One Division overcame this problem by providing taxi vouchers. Parking problems were noted in one urban division. Geographical isolation was mentioned as a barrier in one rural area, particularly for those living on rural properties. However access was not an issue at the other rural site where groups were run at local community venues. All Intervention Officers reported finding it difficult to organise session times that suited the majority of participants. They highlighted the need for flexible arrangements, including running sessions at night and at weekends, and offering individual sessions and telephone follow up for those unable to attend particular group sessions.

Intervention Officers also identified a number of individual factors influencing attendance. There was general agreement that people aged 40–50 years were less likely to come because of work commitments, despite the offer of out of hours sessions. Those aged 50 plus were seen to be easier to contact, to have fewer work commitments and to be more motivated because of their health concerns and health problems amongst their family and friends. Two Intervention Officers also observed that very obese participants (BMI > 35) were more likely to drop out of the program. Journal notes from one Intervention Officer suggest that the exercise component of the sessions may have been a deterrent for these individuals.

Finally, a number of social factors were identified as important in influencing attendance rates. One Intervention Officer noted the value of allowing partners to attend. A rural Intervention Officer reported that attendance was better in smaller communities as people knew each other and the group provided a forum for social interaction. Group leader facilitation skills were also important including encouraging group interaction and participation of each group member, building trust and rapport with individuals and creating a comfortable group atmosphere. Having group leaders who were not seen as ‘experts’ was helpful as they were considered fellow participants in the program.

## Discussion

This mixed methods study provides important new insights into the factors influencing patient attendance at lifestyle intervention programs for the prevention of chronic disease. Over a third of individuals referred to the programs in this study did not attend any sessions, and a further small proportion (4.1%) attended less than half. However, the majority of those who attended initially continued to do so. Individuals who were older, did not work and had higher levels of psychological distress were significantly more likely to attend, while work commitments or problems with accessing the program were seen as important obstacles. GP/practice endorsement of the program and encouragement from group facilitators promoted attendance, along with flexibility arrangements including providing sessions outside of working hours.

Thus the LMP was most strongly taken up by non-working individuals, most of whom were retired. Conflicts with work schedules has been recognised as a reasons for not attending health education programs [39-43], although one study of patients with existing CVD reported greater participation by employed individuals [44]. There was also a suggestion from Intervention Officers that older individuals were more motivated to attend because they saw themselves as more susceptible to ill health, although age was not an independent predictor of attendance in the quantitative analysis. The challenge is to develop lifestyle programs that better engage individuals who are working. This could include lifestyle programs run through workplaces, internet based programs or telephone counselling. While such approaches have been shown to be effective in promoting lifestyle change [45-50], again little is known about the reach of such programs and levels of participation.

Interestingly, those with higher levels of psychological distress were more likely to attend the lifestyle program. The social interaction provided by the group may have been a motivator to attend for those with higher levels of distress. Previous studies examining associations between psychological distress and use of health service have shown conflicting results, with some studies reported increased use of primary health care services for those with high psychological distress [51,52], while others have shown that psychological distress is associated with higher rates of drop out from cardiac rehabilitation programs [53]. Further research is warranted to explore the associations between psychological distress and attendance and use of preventive health services.

Having GPs and practice nurses endorse the program was seen to encourage participation, and practices that were linked with allied health practitioners and had written preventive care protocols were more successful in promoting attendance. This suggests that practices should be briefed about their role in promoting the program, and given timely feedback following the program to encourage further referral and uptake [54], and that this may be assisted by encouraging strong relationships with allied health professionals and a more formalized approach to risk factor management within the practice. Involving group facilitators in enrolling participants may also improve attendance rates.

A number of access problems were identified. This is in line with previous research on poor attendance at health education programs [18,41,42,55]. Access could be improved by running programs in community venues with good public transport links and parking facilities, and through the use of outreach programs such as telephone/internet based programs for rural and remote areas. However, these latter options do not provide the social interaction found in a group program, and may consequently be less appealing. The study also highlights the importance of flexibility in program delivery including providing sessions in the evening and weekends and offering individual intervention and telephone follow up to boost participation rates.

Interestingly, there was no association between the participants’ health risk profile or readiness to change at baseline and attendance rates at the program. This may be because GPs were more likely to refer high risk individuals who were ready to make lifestyle changes. A separate analysis has revealed that, consistent with the study protocol, individuals with elevated BMI, physical inactivity and who were in contemplation/preparation/action stages of change for physical activity were more likely to be referred [56]. The lack of association between participant’s stage of change and program participation may also reflect the fact that stages of change are not static categories and that individuals can shift between stages over a relatively short period of time. There was also no relationship with consumer’s health seeking behaviour as measured by the screening tool developed by Maibach et al. [33]. All our participants were categorised as ‘passive’ with regard to their health orientation, and their participation was not related to their degree of independence in health decision making. This is in contrast to the Intervention Officers’ perceptions that GPs’ endorsement encouraged attendance. It may be that the tool to measure consumer health information preferences is not valid in Australia, or that other factors were more important in determining participation.

Our findings suggest the value of the social interaction and support provided in a group program for encouraging attendance. Evidence also suggests that a group approach may be more effective in promoting weight loss than individual intervention [57]. The findings highlight the importance of group leaders having good facilitation skills in order to create a comfortable and inclusive group atmosphere. The non–expert role of the Intervention Officers in this study was reported to facilitate engagement and rapport building with participants, suggesting the potential value in lay or non professionally led programs.

This study focused on factors influencing participation in LMP amongst those eligible to attend (ie those completing a health check and referred by their GP). It is important to acknowledge however, that only around 30% of those initially invited to participate in the study agreed to do so. Factors influencing enrolment in diabetes and CVD prevention programs are an important and related issue. Many efficacy and replication trials have not provided any information on enrolment rates amongst eligible participants [12,58-65]. In other trials the proportion of eligible participants who agreed to enroll has varied widely from a third to 100 percent [8,9,18,66-71]. Little is known about factors influencing enrolment in these programs. As with this study, it is often difficult to examine predictors of enrolment as ethics requirements prevent information being collected on individuals invited who decline to participate. In order to improve the reach of these programs, factors influencing both enrolment and program completion are important areas for ongoing research.

This study had a number of limitations. We did not conduct follow up interviews with participants who were low attenders of the LMP, although we did ask for reasons for non attendance in the 12 month participant survey. Qualitative interviews with participants who are invited but do not attend lifestyle programs could elicit further insights into factors influencing participant engagement and the way programs could be modified to improve future attendance.

## Conclusion

Over a third of participants referred to the LMP in this study did not attend any sessions and 41% were considered to be low attenders (attending less than half of the planned sessions). Barriers to attendance identified through quantitative and qualitative findings mainly related to external factors including work commitments and poor physical access to the program rather than individuals’ motivation to change or health risk profile. Participants who did not work and those with higher psychological distress were more likely to attend the LMP. Factors considered to facilitate attendance included GP/Practice and Intervention Officer endorsement of the program and encouragement to attend, as well as flexibility in program delivery.

## Competing interests

Authors declare that they have no competing interest in the conduct of this study.

## Authors' contributions

All authors contributed to the conception and design of the study and interpretation of data. RL and UJ contributed data analysis, RL wrote the first draft of the manuscript. All authors contributed to revising the manuscript and approved the final draft.

## Pre-publication history

The pre-publication history for this paper can be accessed here:

http://www.biomedcentral.com/1472-6963/13/201/prepub
